# A Novel Role for Relaxin-2 in the Pathogenesis of Primary Varicosis

**DOI:** 10.1371/journal.pone.0039021

**Published:** 2012-06-21

**Authors:** Julia Adams, Sarah Schott, Arno Bern, Matthias Renz, Kristian Ikenberg, Claus Garbe, Christian Busch

**Affiliations:** 1 Department of Dermatology, University of Tuebingen, Tuebingen, Germany; 2 Department of Gynecology, University of Heidelberg, Heidelberg, Germany; 3 Practice Clinic of Phlebology, Tuebingen, Germany; 4 Institute of Pathology, UniversitaetsSpital, Zuerich, Switzerland; Morehouse School of Medicine, United States of America

## Abstract

**Background:**

Varicose veins affect up to 40% of men and up to 51% of women. The pathophysiology of primary varicosis is poorly understood. Theories ranging from incompetence of the venous valves to structural changes in the vein wall have been proposed.

**Methodology/Principal Findings:**

We analyzed the functional state of the intramural smooth muscle cells (n = 14 pairs matched for age and gender) and the expression of relaxin-2 and its receptors RXFP1 and RXFP2 in samples of varicose and healthy great saphenous veins (GSV) (n = 21 healthy GSV; n = 46 varicose GSV). Relaxin-2 and RXFP1 contents were determined in tissue samples (n = 9 samples per group). Pharmacological analyses were performed in a perfusion chamber. Morphometric determination of the nuclear size of the smooth muscle compartment yielded no significant difference in varicose GSV in comparison with the healthy controls. Relaxin-2 and its receptors were expressed in the muscular layer, endothelial cells and in blood vessels contained in the vein wall. Immunohistochemical expression of relaxin-2, RXFP1 and RXFP2 was significantly decreased in varicose GSV. Relaxin-2 and RXFP1 measured by ELISA and Western Blot were decreased in varicose GSV (relaxin-2 ELISA healthy vs. varicose GSV: 12.49±0.66 pg/mg versus 9.12±3.39 pg/mg of total protein; p = 0.01; Student's T-test). Contractions of vein samples induced by cholinergic or adrenergic stimulation were antagonized by relaxin-2.

**Conclusions/Significance:**

We report that relaxin-2 and its receptors RXFP1 and RXFP2 are expressed in GSV and that their expression is significantly decreased in varicose GSV. Further, we were able to demonstrate a functional pharmacological relaxin-2 system in varicose GSV. Our results suggest a novel role for relaxin-2 in the pathogenesis of primary varicosis, rendering relaxin-2 a novel possible pharmacological agent for the treatment of this widely prevailing venous disease.

## Introduction

Varicose veins affect up to 40% of men and up to 51% of women. Western lifestyle, hormonal changes, age, female gender, parity, adipositas and standing occupation are risk factors associated with varicosis [1]–[3]. Little is known about the pathophysiology of primary varicosis. Theories have been proposed, such as functional abnormalities in the valve structures, biological (matrix metalloproteinase (MMP) expression) or morphologic changes of the vein wall (hypertrophy/loss of smooth muscle cells) and extra-cellular matrix organization (collagen and elastin content). However, conflicting data have been reported on all these issues [4], [5]. Recently, human thymic smooth muscle cells were analyzed for MMP-2 expression and gelatinase activity under experimentally-induced cyclic stretch as a surrogate for smooth muscle cells from the tunica media of the GSV [6]. A gender-specific expression of estrogen and progesterone in healthy and varicose GSV was reported [7].

Relaxin belongs to the insulin-like growth factor family [8]. Three different relaxin genes have been found in humans, known as RLN1, RLN2, and RLN3. Relaxin exerts its actions via its leucine-rich-repeat-containing G-protein coupled receptors (GPCR) RXFP1 and RXFP2. Upon receptor binding, intracellular relaxin signaling is mediated by Smad, MAPK, ERK, and PI3 kinase pathways. As “pregnancy hormone”, relaxin promotes elongation of the interpubic ligament in mammals, inhibits spontaneous contractions of the uterine myometrium in guinea pigs and promotes cervical softening in several species [9]. It is produced in small quantities within the male reproduction tract and has been detected in the human prostate. Further, relaxin is involved in fibrosis, wound healing, cardiac protection, allergic responses and cancer [9].

Of particular interest are the anti-fibrotic and the vasodilatory effects of relaxin. Relaxin increases MMP-induced collagen synthesis and decreases the actions of the tissue inhibitors of MMPs. Human relaxin-2 reverses pathological collagen accumulation in numerous animal models of induced fibrosis [10]. Vasodilatory effects are mediated by promotion of nitric oxide (NO) in addition to antagonism of the vasoconstricting actions of endothelin-1 and angiotensin II [11].

In this study we analyzed the functional state of smooth muscle cells of the tunica media in control and varicose GSV by nuclear morphometry and studied a possible role for relaxin-2 in the pathogenesis of varicosis by comparing the expression of relaxin-2, RXFP1 and RXFP2 in control and varicose GSV. Moreover, pharmacologic stimulation of samples of GSV was performed to characterize the function of relaxin in GSV.

## Results

### Quantitative morphometric analysis of nuclear size of smooth muscle cells

**Figure 1:**
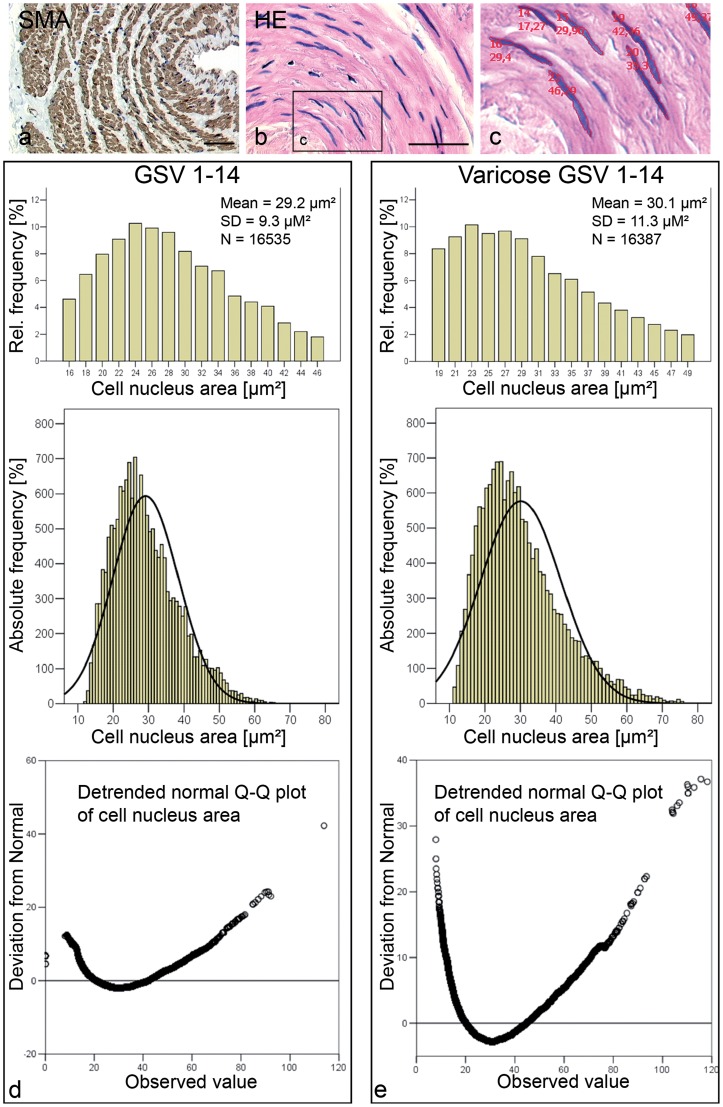
Morphometry of smooth muscle cell nuclei of the tunica media of GSV. (A) Representative cross-section of a GSV. Smooth muscle cell compartment is visualized by smooth muscle actin staining. (B, C) Morphometry was conducted on HE stained slides (using Zeiss Axiovision). Statistical analysis of nuclear measurements of paired healthy (D) and varicose (E) GSV specimens demonstrating no significant difference of nuclear size in varicose GSV compared to healthy GSV.

We investigated the functional state of smooth muscle cells in the tunica media by measuring the nuclear size as quantitative parameter. The projection area of the nucleus was taken as direct parameter to estimate the volume of the nucleus, which correlates with the functional state of the cell. Smooth muscle cells were visualized in SMA staining for better contrasting and clear identification (Fig. 1A). From the parallel HE stained slide, digital images of 15 to 20 optical fields (magnification: 40x) were recorded for measurement (Figs. 1B, C). An average of 1176 nuclei per GSV were measured (Table 1 and Table S1). The standard deviation (SD) in the measurement per patient is not only due to the measuring error, but also due to the variation in the location of the nuclei in the sections. This variation in the projection of the nuclei was assumed to be identical in the specimens due to the high number of measured nuclei. The average of the nuclear areas in the group of control GSV was 29.2±9.3 μm^2^ compared to 30.1±11.3 μm^2^ in the group of varicose veins (Figs. 1D, E). The difference in nuclear size between healthy and varicose GSV was not significant; however, the SD of the nuclear areas was higher in the group of varicose GSV.

**Table 1:** Cell nucleus areas of 14 pairs of healthy and varicose GSV samples.

Pair no.	GSV		Varicose GSV	
	Cell nucleus area [µm^2^]	n	Cell nucleus area [µm^2^]	N
1	31,04±9,90	1157	38,73±12,10	978
2	27,85±8,64	967	35,23±9,64	875
3	35,70±9,57	697	37,54±15,03	588
4	28,73±8,30	1239	35,97±9,32	966
5	30,25±9,12	759	46,65±11,72	1310
6	29,67±8,55	1364	25,87±7,19	1170
7	38,45±10,69	962	31,32±9,02	1070
8	30,04±8,25	913	21,86±6,47	1191
9	33,57±8,13	1337	30,06±8,27	1508
10	26,49±8,27	1372	30,12±8,87	1350
11	26,69±8,38	1489	21,09±5,93	1559
12	24,27±6,81	1822	26,41±6,04	1249
13	28,46±8,41	1148	21,23±5,28	1363
14	25,43±7,11	1309	30,05±7,24	1210
Mean	29,17		30,12	
SD	9,25		11,33	

### Immunohistochemical localization of relaxin-2, RXFP1 and RXFP2; measurement of relaxin-2 and RXFP1 proteins in samples of control and varicose GSV

**Figure 2:**
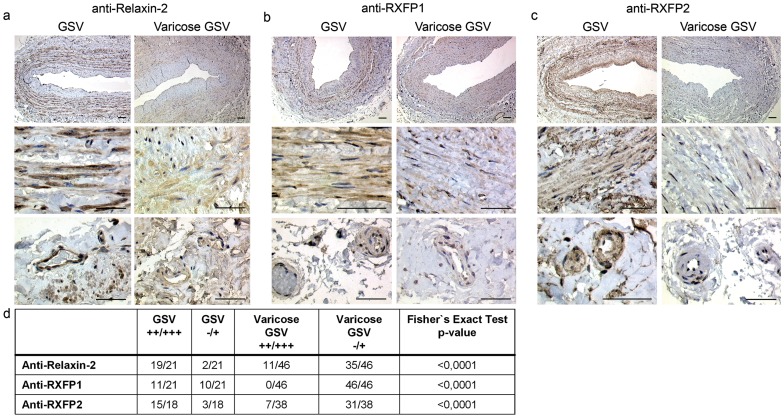
Immunohistochemical detection of relaxin-2 and its receptors RXFP1 and RXFP2 in healthy and varicose GSV. (A) Anti-relaxin-2-staining strongly labels endothelial cells, the tunica media and vasa vasorum of the adventitia in healthy GSV and less intense in corresponding compartments of varicose GSV. (B, C) Both RXFP1 and RXFP2 show decreased staining intensity in similar compartments in varicose GSV compared to healthy GSV. (D) Statistical evaluation of staining score.

The expression of relaxin-2, RXFP1 and RXFP2 was analyzed in samples of 21 healthy (control) and 46 incompetent (varicose) GSV (Table S2) by immunohistochemistry. In healthy GSV, relaxin-2 and its receptors were strongly expressed in endothelial cells, in smooth muscle cells of the tunica media, and in vasa vasorum of the adventitia (Figs. 2A, B, C). In varicose GSV, the principal expression pattern of relaxin-2, RXFP1 and RXFP2 was similar to the healthy control veins (Figs. 2A, B, C). Evaluation of proximal and distal samples of the same varicose GSV showed no differences in expression of relaxin-2, RXFP1 and RXFP2 (not shown). However, the expression levels (intensity) of relaxin-2, RXFP1 and RXFP2 were significantly lower in varicose GSV (Fig. 2D; p<0.01; Fishers's Exact Test, two-tailed). For relaxin-2 and RXFP2 no influence of age or gender on protein staining intensity was found. For RXFP1 we detected an age-independent significant difference of staining intensity in the healthy control GSV samples when comparing male and female donors. Females: 8/10 with strong expression of RXFP1; males: 2/11 with strong expression of RXFP1 (p<0.01; Fisher's Exact Test, two-tailed).

**Figure 3:**
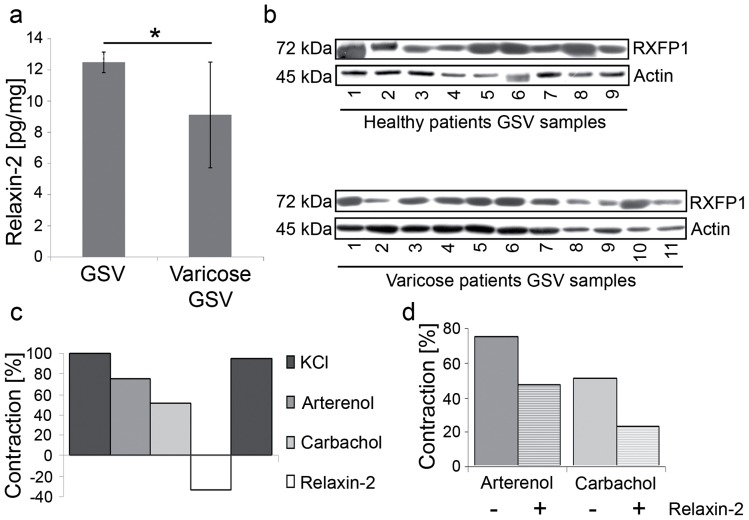
Expression of relaxin-2 and its receptor RXFP1 in surgical samples of healthy and varicose GSV, and pharmacological stimulation. (A) Relaxin-2 content is decreased in varicose compared to healthy GSV, determined by ELISA (9.12±3.39 pg/mg versus 12.49±0.66 pg/mg of total protein; p = 0.01; Student's T-test). (B) Western Blot detection of RXFP1 in healthy and varicose GSV. RXFP1 bands are less pronounced in the varicose GSV samples. (C) In vitro pharmacology of surgical samples of GSV. Pulse-stimulations with KCl (kaliumchloride), arterenol (norepinephrinehydrochloride) and carbachol (carbamylcholine) induce contractions of the GSV specimens; relaxin-2 relaxes the specimens. The first KCl contraction was defined as 100% maximum contraction. Arterenol: 76%, carbachol: 51%, relaxin-2: −34% of maximum KCl contraction. (D) Constant perfusion with relaxin-2 partly antagonizes the contractions induced by pulse-stimulations with arterenol and carbachol: Arterenol: 76 to 47% (reduction of 29% of KCl maximum concentration), carbachol: 51 to 23% (reduction of 28% of KCl maximum concentration.

To verify the immunohistochemical data, relaxin-2 content was determined using an ELISA assay in samples of 9 healthy and 9 varicose GSV (4 female and 5 male donors in each group). Here too, the relaxin-2-level was significantly lower in varicose GSV compared to the healthy controls (9.12±3.39 pg/mg versus 12.49±0.66 pg/mg of total protein; p = 0.01; Student's T-test) (Fig. 3A). Western Blot analyses in samples of 9 healthy (4 female, 5 male donors) and 11 varicose GSV (4 female, 7 male donors) also demonstrated a reduced expression of RXFP1 in the varicose GSV samples (Fig. 3B).

### Ex-vivo pharmacology of KCl, arterenol, carbachol and relaxin-2 in surgical samples of varicose GSV

To determine a functional relaxin-2-system in GSV, we performed pharmacological testing with adrenergic and cholinergic stimuli to assess the contraction capacity of the smooth muscle compartment and a possible modification thereof by relaxin-2. In total, 7 different specimens of each GSV (from 2 female donors) were used for the pharmacological stimulations with different drugs. Figures 3C and D depict the results of an exemplary series of stimulations. As expected, KCl induced a maximum contraction of the vein wall (defined as 100% maximum contraction). Adrenergic and cholinergic stimulations induced contractions that were less pronounced than the KCl contraction (Arterenol: 76%; Carbachol: 51% of KCl maximum concentration). Pulse stimulation with relaxin-2 induced a relaxation of the tissue specimens (corresponding to −34% of KCl maximum concentration; Fig. 3C). A final stimulation with KCl was re-performed, demonstrating a sustained vitality and contractability capacity of the tissues (94% of original maximum concentration). The pharmacological stimulations were repeated under constant perfusion with relaxin-2. Relaxin-2 partly antagonized the adrenergic and cholinergic contractions (Arterenol: 76 to 47% (reduction of 29% of KCl maximum concentration); Carbachol: 51 to 23% (reduction of 28% of KCl maximum concentration; Fig. 3D).

## Discussion

Distensibility of the vein wall is controlled by smooth muscle cells, collagen and elastin. The smooth muscle compartment of the tunica media is responsible for wall tone, which is controlled by autonomic nerves and circulating stimulants. Passive tone is provided by collagen and elastin. Changes in collagen, elastin and smooth muscle contents in varicose GSV leading to reduced contractibility and compliance have been reported [4]. Relaxin mediates smooth muscle relaxation and collagen turnover. Since both aspects play important roles in the development of primary varicosis, the aim of the present study was to determine the expression pattern of relaxin-2 and its receptors RXFP1 and RXFP2 in healthy and varicose GSV.

To assess the functional state of smooth muscle cells we used nuclear size measurements as a quantitative parameter. Measurement of nuclear size in histological sections is a reliable method for the quantitative determination of the functional state of endocrine organs and was used to elucidate the hypophyseal gonadal and adrenal axis [12], [13] and the smooth muscle cells in uterosacral ligaments of patients with and without pelvic organ prolapse [14]. Since apoptosis is not detectable in the muscular compartment of varicose GSV [15], nuclear size changes known to occur during apoptosis had not to be taken into account. We observed no differences of the cell nucleus area of smooth muscle cells in healthy and varicose GSV. The mean nuclear size had a higher standard deviation in the varicose group. Whether this possibly reflects the disorganization of the muscular layer previously described for varicose veins [2], [3] remains to be elucidated.

It is not yet clear if primary varicosis is caused by endocrine factors, inherent or acquired structural defects or external factors such as mechanical stress. Influences of the sex hormones estrogen and progesterone on tissue metabolism in veins have been suggested [7], [15], however, a functional relevance for varicosis could not be established. Our results revealed that the expression of relaxin-2 and RXFP2 was reduced in the varicose GSV independent of sex and age, suggesting a muted relaxin-system leading to reduced short-term functionality of the smooth muscle compartment and possibly responsible for long-term fibrotic accumulation of collagen in the varicose vein wall. For RXFP1 expression we found an age-independent, significantly increased expression in female healthy control GSV samples when compared to healthy samples from male donors.

Functional pharmacology of life surgical samples under the microscope confirmed the adrenergic and cholinergic nature of the smooth muscle compartment of the GSV [1]. Moreover, it demonstrated for the first time that relaxin-2 antagonized the adrenergic and cholinergic contractions, suggesting that relaxin-2 might be also involved in short-term, active fine-tuning of the active tone of the vein wall in addition to the long-term, passive tone set up by collagen and elastin turnover. Relaxin-2 exerts its short-term effects via NO induced in the smooth muscle cell or secreted as relaxing factor from the endothelium [16]. The effect of relaxin-2 on the sensitivity toward arterenol is well explained by interference with smooth muscle contraction via NO production and increased cGMP levels.

Limitations of the study were (i) the age difference of male and female patient donors, which however had no effect on the immunohistochemical expression of relaxin-2 and its receptors; (ii) the imperfect matching of four out of the fourteen matched pairs used for nuclear morphometry; (iii) the relatively low number of healthy (control) GSV specimens (n = 9) analyzed in ELISA and Western blot, and (iiii) the lack of pharmacological data on healthy GSV samples. Therefore further studies are required to back up the presented and in part preliminary data suggesting a pathophysiological role for relaxin-2 in primary varicosis.

Together, we were able to demonstrate for the first time that relaxin-2, RXFP1 and RXFP2 are less expressed in varicose GSV compared to healthy GSV and that relaxin-2 antagonizes the adrenergic and cholinergic contractions of the smooth muscle compartment in GSV. Considering the smooth muscle-relaxing and anti-fibrotic actions of relaxin, our findings strongly suggest that the reduced expression of relaxin-2 in varicose GSV might play a pivotal role in the pathogenesis of primary varicosis and identify relaxin-2 as the first potential agent for pharmacological treatment of primary varicosis.

## Materials and Methods

### Recruitment of patients and tissue specimens

Patients <18 years, with post-menopausal hormonal replacement therapy, pregnancy, severe chronic (autoimmune) diseases (e.g. diabetes mellitus, cardiac insufficiency, lupus erythematodes), stage IV tumor patients and patients with post-thrombotic reflux in the GSV were excluded from this study. As healthy controls, surplus GSV were obtained from two different sources: 1. Patients undergoing elective coronary bypass surgery at the Department of Thorax, Vascular, and Cardiac Surgery, University of Tuebingen, Germany (n = 13). 2. Melanoma patients diagnosed with micrometastasis or single melanoma cells detected in the sentinel lymph node biopsy undergoing radical inguinal lymph node dissection (n = 8). GSVs of control patients were assessed by duplex ultrasonography for reflux before operation by trained technicians. None of the included control patients had duplex ultrasonographic, clinical or intraoperative signs of venous insufficiency or varicosis (C0–C1).

Incompetent (varicose) GSV were obtained from patients with primary varicosis (n = 26) during saphenectomy by stripping either at the Department of Dermatology, University of Tuebingen, or at the praxis clinic Dr. A. Bern/Prof. M. Moehrle at Tuebingen, Germany. Preoperative evaluation of vein status was obtained by duplex ultrasonography by trained technicians, showing retrograde flow all along the venous GSV-axis (sapheno-femoral junction, long saphenous vein and perforating veins) (C2–C4). None of the varicosis patients presented with venous ulcers (C5–C6). In summary, the varicose patients were C_2–4_E_p_A_s/p_P_r_. The healthy control patients were C_0–1_E_n_A_n_P_n_. For histological and immunohistochemical analysis, 20 additional samples of paraffin embedded varicose GSV derived from GSV stripping operations from patients with primary varicosis (C_2–4_E_p_A_s/p_P_r_)were retrieved from the files of the Department of Pathology, University of Tuebingen to increase the number of samples. Details (age and gender) of the patients included in this study: Control patients: female: n = 10, mean age: 74 years; male: n = 11, mean age: 66 years; combined mean age: 70 years. Varicose patients: female: n = 30, mean age: 57 years; male: n = 11, mean age: 50 years; combined mean age: 53 years (see also Table S2).

### Removal of saphenous veins and processing

About 5–10 cm samples of GSV of both groups were obtained during surgery. In some cases entire varicose GSV were obtained after stripping operation. From these veins proximal and distal samples were used. Tissue specimens were cut into three sections: One third was transferred into 4°C PBS and directly transported to the laboratory. For protein extraction, the second third was snap-frozen and stored in liquid nitrogen until protein extraction. For immunohistochemical analysis the last third of the tissue was immediately fixed in 4°C buffered paraformaldehyde for 24 h. After rinsing, the samples were embedded in paraplast using a tissue processor (Shandon Citadel, Thermo Fisher Scientific, Dreieich, Germany). Serial sections of 3 μm were cut (longitudinal and transversal to the course of the veins), mounted onto polylysin-coated slides and stained with Hematoxylin and Eosin (H&E) for histological evaluation. For histological and immunohistochemical analysis, additional samples of paraffin embedded varicose GSV were retrieved from the files of the Department of Pathology, University of Tuebingen, Germany. For protein extraction, a pre-cooled mortar and pestle was used. The tissue samples were minced in liquid nitrogen, the frozen powder was dissolved in RIPA buffer (Sigma, Munich, Germany) 20 min on ice for cell lysis and protein extraction, followed by 15 min centrifugation at 4°C. The supernatant was frozen until further evaluation. Protein content was determined by Bradford assay.

### Histological staining and immunohistochemistry

The primary antibodies used in this study were anti-human relaxin-2 (1∶300, rabbit, polyclonal, Immundiagnostik AG, Bensheim, Germany), anti-relaxin receptor 1 (anti-RXFP1, 1∶50, rabbit, polyclonal, GenWay, San Diego, CA, USA), anti-relaxin receptor 2 (anti-RXFP2, 1∶50, rabbit, polyclonal, Genway), and smooth muscle α-actin (SMA, 1∶100, IgG2a, kappa, clone 1A4, DAKO, Hamburg, Germany).

Immunostaining was carried out using standard protocols. Briefly, sections were de-waxed in xylene, and rehydrated in grades of industrial methylated spirit and distilled water. Endogenous peroxidase activity was then quenched using hydrogen peroxide for 10 min. After two washes in de-ionized H_2_O and phosphate-buffered saline (PBS), sections were blocked for 15 min with PBS containing 3% bovine serum albumin (BSA), and then further blocked for 30 min with normal serum to minimize non-specific reactivity. Further blocking was performed with avidin-biotin blocking solution. The slides were incubated overnight at 4°C with the primary antibody in a humidified chamber. Sections were incubated with the secondary antibody, then with peroxidase for 30 min each. Bound antibodies were visualized with 0.5% diaminobenzidine (DAB) in 0.5 mol/l tris-HCl pH 7.4, and 0.01% hydrogen peroxide according to the manufacturer’s instructions (Dako, Germany). After washing with distilled water the slides were counterstained for 2–3 min with Mayer's Hemalum. Sections were dehydrated through graded ethanol, cleared with xylene and permanently mounted in DEPEX. Negative control sections were produced by omission of the primary antibody and did not show any staining, while the positive control slides (human endometrium and myometrium) were stained with the primary antibody, and there was no background reaction. The positive staining controls are depicted as Figure S1A. For immunohistochemical analyses, paraffin sections of samples of 21 control GSV and 46 varicose GSV were used (Table S2).

### Image analysis, nuclear size morphometry and evaluation of immunohistochemistry

Digital images were acquired using an Axioplan 2 microscope (Carl Zeiss, Jena, Germany) with a mounted digital camera (Zeiss AxioCam color, Carl Zeiss) and analyzed with image analysis software (Zeiss Axiovision 4.2, Carl Zeiss; Adobe Photoshop CS2, Adobe Systems GmbH, Munich, Germany). To assess the functional state of the smooth muscle cells the nuclear area was measured in H&E sections. The smooth muscle cell nucleus has a rod shaped form with blunted edges. The computer-assisted morphometry allows direct determination of the projection area of the nuclei. Thus, the calculation of area or volume on the basis of assumed geometric forms, and determination of diameters were not necessary. Due to the orientation of the specimen in the paraffin blocks the circular part of the tunica media was in general cut in longitudinal direction. Cross-sectional areas were excluded from the nuclear measurement. Cell nucleus size was measured on the H&E slides because of better contrasting of the nuclei. Morphometry of longitudinally cut smooth muscle cell nuclei of the tunica media in cross sections of the veins was performed as described previously in detail for smooth muscle cells in samples of human sacrouterine ligaments [14]. For morphometric measurements, control and varicose GSV were matched to 14 pairs (Table S1) according to age and gender. In 10/14 pairs the matching was successful; the remaining four pairs were matched according to age (one pair), according to gender (one pair) or according to the diagnosis alone (two pairs). The measuring person (JA) was blinded to the clinical diagnosis, and slides were measured in a random order. Slides stained for relaxin-2, RXFP1 and RXFP2 were analyzed blinded to the clinical diagnosis in every case. A semi-quantitative staining score was established (0/+/++/+++; see Figure S1B), and all slides were classified accordingly.

### ELISA

For detection and quantification of relaxin-2, a commercially available Relaxin-2 ELISA KIT (Immundiagnostik AG) was used according to the manufacturer's instructions. Briefly, the assay utilized the “sandwich” technique with two selected polyclonal antibodies that bind to human relaxin-2. Assay standards and pre-diluted GSV samples were added into the wells of a microplate coated with a high affine polyclonal anti-human relaxin-2 antibody. During the first incubation step (4°C, 16 h), relaxin-2 was bound by the immobilized antibody. Then a detection antibody, biotin-labeled anti relaxin-2, was added for 2 h at 4°C. Afterwards a peroxidase conjugate was added to form a “sandwich” of capture antibody – human relaxin-2-detection antibody–peroxidase-conjugate. Tetramethylbenzidine (TMB) was used as peroxidase substrate (incubation: 1 h at 4°C). Acidic stop solution was added to terminate the reaction. The color changed from blue to yellow. The intensity of the yellow color is directly proportional to the concentration of relaxin-2. A dose response curve of the absorbance unit (optical density, OD at 450 nm) vs. concentration was generated using the values obtained from the standard. Relaxin-2 present in the GSV samples was determined directly from this curve.

### Western blot

15 µg protein of each GSV sample (see above) were subjected to SDS–PAGE and transferred to polyvinylidene difluoride membranes. After blocking in PBS/0.1% Tween-20/5% dry milk for at least 1 h, the blots were probed overnight with primary antibodies in PBS/0.1% Tween-20/5% dry milk, washed with PBS for 3×10 min and incubated with secondary biotin-conjugated antibody (Dako, Grostrup, Denmark). After washing with PBS for 3×10 min, the Streptavidin–Alkaline–Phosphatase conjugate (Roche) was used for the detection of biotin-labeled secondary antibody. The membrane was immersed in CDP-Star solution (Roche) for 10 min and then exposed to X-ray film (Eastman Kodak, Rochester, NY). The following primary antibodies were used: anti-RXFP1 (1∶1000, Santa Cruz Biotechnology, Santa Cruz, CA, USA) and anti-β-actin (1∶1000, Cell Signaling, Frankfurt, Germany).

### Pharmacology: Equipment, application of drugs, data acquisition and evaluation

Pharmacological stimulation was performed on two samples of varicose GSV (female donors, age 41 and age 54) to determine whether adrenergic, cholinergic and relaxin-2 stimulation were able to induce contraction or relaxation of the muscular compartment. The central instrument was a stereomicroscope (Stemi IV, Zeiss) equipped with a digital camera (Olympus). The open perfusion chamber consisted of a rubber gasket (inner diameter 0.8 cm) glued to a microscope slide and perforated with three syringes, two for inlet (for perfusion medium without and with relaxin-2, respectively) and one for outlet. The temperature of 37°C was controlled via a heated object stage and a heating plate (Zeiss), onto which coils of the afferent perfusion tubes and the tube for gas supply (carbogen, 95% O_2_ and 5% CO_2_) were fixed under styrofoam insulation. The heated and moistened gas stream was directed on the surface of the perfusion medium in the chamber. Continuous perfusion with carbonate buffered Hanks solution +2.5% 1 M Hepes was maintained by a peristaltic pump (Type ISM597A, Ismatec SA, Zuerich, Switzerland) and afferent and efferent silicon tubes. Small straps of longitudinally and circularly cut GSV and cross sections were used after careful removal of endothelium and the tunica adventitia with forceps and bent scissors. The tissues were carefully placed into the perfusion chamber.

The following drugs were used for bolus stimulation: KCl (kaliumchloride, 1 mM, Fresenius Kabi, Bad Homburg, Germany), arterenol (norepinephrine hydrochloride, noradrenaline, 10^−3^ M, Sanofi-Aventis, Frankfurt, Germany), carbachol (carbamylcholine, 6.6 M, Sigma), recombinant human relaxin-2 (10 µg/ml, Immundiagnostik AG). After each series of stimulation (KCl, norepinephrine hydrochloride, carbachol, relaxin-2) a final stimulation with KCl was re-performed to assure viability/contractability of the tissues. As negative control perfusion with HBSS was performed.

Drugs were applied by aspiration via the influx tube. During relocation of the afferent silicone tube the peristaltic pump was switched off for about 5 sec to avoid bubble formation. Pulse stimulation was performed by aspiration of 200 µl of the respective drug solution via the afferent silicon tube. The drug reached the chamber within 75 sec through the afferent silicone tubes (one for solutions without and one for solutions with relaxin-2) with an inner diameter of 0.44 mm, a length of 1.3 m including the coils on the heated microscope stage, a volume of 198 µl, and a flow rate of 140 µl per minute. The volume of the chamber was 500 µl. The effective maximum drug concentration during the pulse stimulation was estimated by assuming a dilution factor of 0.5 for the inflowing drug and an elimination factor of another 0.5 due to efflux with the same rate. Thus in the pulse stimulation experiments the effective drug concentration was one order of magnitude lower than in the 200 µl applied. Between consecutive pulse stimulation sequences the transfer of the 700 images of a sequence to the database took about 3 min during which the probe was rinsed with medium. In addition to the pulse stimulations, the experiments were repeated under constant perfusion with relaxin-2 (100 ng/ml in HBSS +11 mM glucose) instead of the medium described above.

For each pharmacological stimulation a sequence of 700 images with a resolution of 1376×1032 pixel and a file size of 1.85 GB was recorded. The time-lapse factor was 0.5 seconds (length of sequence: 5 minutes 50 seconds). For analysis of the tissue contractions/dilations, the dimensions of the tissues (of images 1 and 700 of each stimulation) were measured (length/width of the tissue straps, or diameter of cross-sections, respectively) by using image analysis software (Zeiss Axiovision 4.2, Carl Zeiss). The change of dimension after the first KCl stimulation was defined as 100% contraction, and the subsequent changes of dimension after the other stimulations of the same tissue sample were calculated as percentage of the 100% primary KCl contraction.

### Statistical analysis

For the statistical analysis of immunohistochemical evaluation, two groups of samples were clustered: – and + (low expression), versus ++ and +++ (strong expression). Statistical analysis of morphometric data and immunohistochemical evaluation were performed by using Student's T-test, Fisher's Exact Test and Wilcoxon Test. P-values <0.05 were considered as statistically significant. Statistical analysis was performed by SPSS Version 11.5 (SPSS GmbH Software, Munich).

## Supporting Information

Figure S1 **Human uterus control stainings for relaxin-2, RXFP1 and RXFP2, and staining score.** (A) Human endometrium and myometrium was used as positive control for immunohistochemistry. As expected, expression of relaxin-2 and its receptors is restricted to endometrium and myometrium. (B) A four-step staining score was applied to evaluate immunohistochemical expression of relaxin-2, RXFP1 and RXFP2; shown are images of anti-relaxin-2 staining. Scale bars  = 50 µm.
(TIF)

Table S1 **Age and gender of the 14 pairs of healthy and varicose GSV used for nuclear morphometry.**
(DOC)

Table S2 **Age and gender of the donor's of healthy and varicose GSV samples used for immunohistochemical analysis of relaxin-2, RXFP1 and RXFP2-expression.**
(DOC)
