# Supplementary figures and images for: A Novel Role for Relaxin-2 in the Pathogenesis of Primary Varicosis
Source: PLoS One. 2012 Jun 21;7(6):e39021. doi: 10.1371/journal.pone.0039021 (PMC3380868; doi:10.1371/journal.pone.0039021)

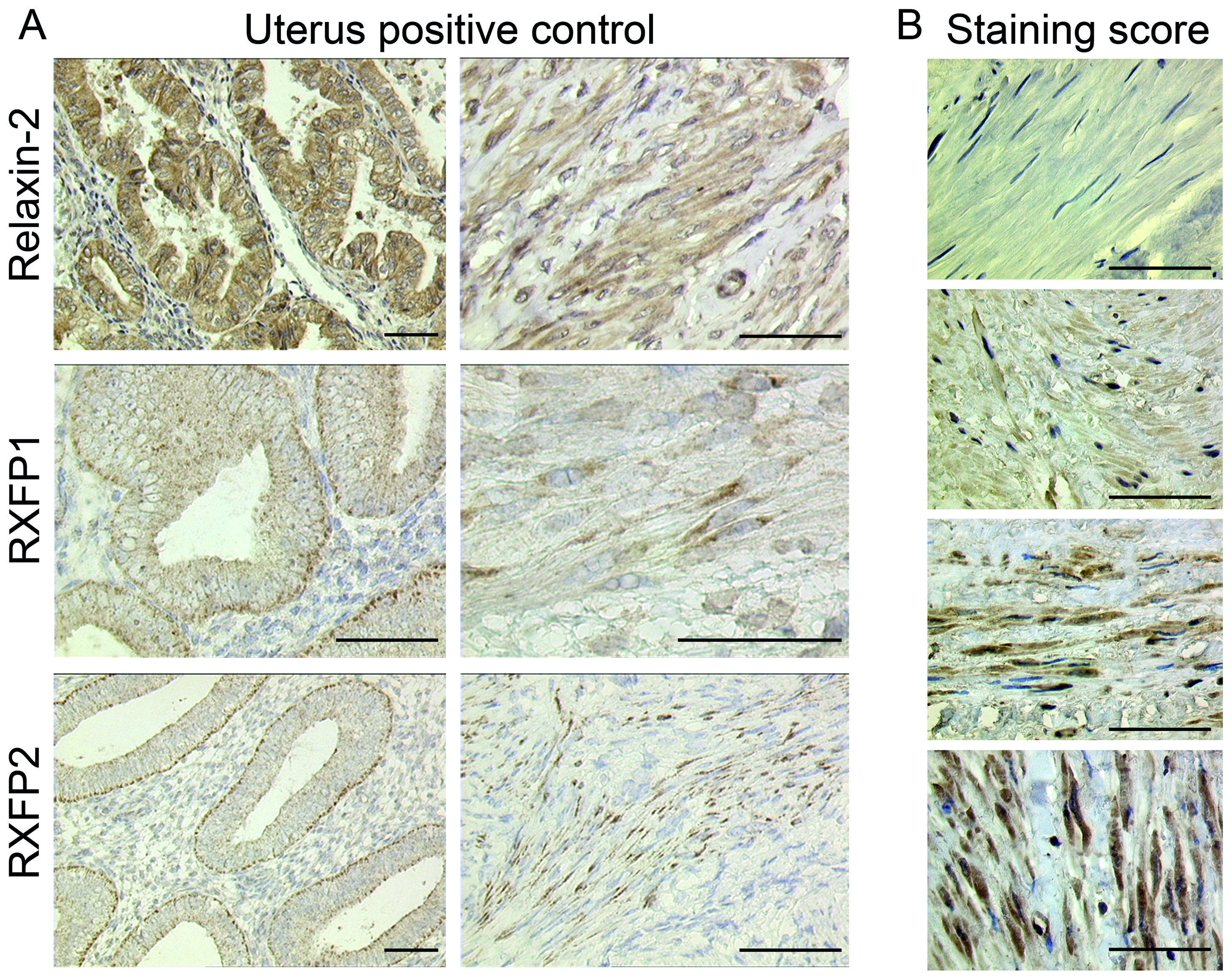

Supplement: Figure S1 — Human uterus control stainings for relaxin-2, RXFP1 and RXFP2, and staining score. (A) Human endometrium and myometrium was used as positive control for immunohistochemistry. As expected, expression of relaxin-2 and its receptors is restricted to endometrium and myometrium. (B) A four-step staining score was applied to evaluate immunohistochemical expression of relaxin-2, RXFP1 and RXFP2; shown are images of anti-relaxin-2 staining. Scale bars = 50 µm. (TIF) [file pone.0039021.s001.tif]
