# Supplementary material for: A Novel Role for Relaxin-2 in the Pathogenesis of Primary Varicosis
Source: PLoS One. 2012 Jun 21;7(6):e39021. doi: 10.1371/journal.pone.0039021 (PMC3380868; doi:10.1371/journal.pone.0039021)
Supplement: Table S1 — Age and gender of the 14 pairs of healthy and varicose GSV used for nuclear morphometry. (DOC) [file pone.0039021.s002.doc]

| Pair no. | Age | Gender | Diagnosis |
| --- | --- | --- | --- |
| 1 | 58 | Male | GSV |
|  | 54 | Male | Varicose GSV |
| 2 | 71 | Male | GSV |
|  | 74 | Male | Varicose GSV |
| 3 | 59 | Male | GSV |
|  | 24 | Female | Varicose GSV |
| 4 | 69 | Male | GSV |
|  | 63 | Male | Varicose GSV |
| 5 | 70 | Male | GSV |
|  | 71 | Female | Varicose GSV |
| 6 | 81 | Female | GSV |
|  | 65 | Female | Varicose GSV |
| 7 | 70 | Male | GSV |
|  | 43 | Male | Varicose GSV |
| 8 | 77 | Female | GSV |
|  | 81 | Female | Varicose GSV |
| 9 | 79 | Female | GSV |
|  | 76 | Female | Varicose GSV |
| 10 | 71 | Female | GSV |
|  | 44 | Male | Varicose GSV |
| 11 | 66 | Female | GSV |
|  | 70 | Female | Varicose GSV |
| 12 | 71 | Female | GSV |
|  | 86 | Female | Varicose GSV |
| 13 | 70 | Female | GSV |
|  | 69 | Female | Varicose GSV |
| 14 | 81 | Female | GSV |
|  | 78 | Female | Varicose GSV |

**Table S1**

Age and gender of the 14 pairs of healthy and varicose GSV used for nuclear morphometry
