# Supplementary material for: A Novel Role for Relaxin-2 in the Pathogenesis of Primary Varicosis
Source: PLoS One. 2012 Jun 21;7(6):e39021. doi: 10.1371/journal.pone.0039021 (PMC3380868; doi:10.1371/journal.pone.0039021)
Supplement: Table S2 — Age and gender of the donor's of healthy and varicose GSV samples used for immunohistochemical analysis of relaxin-2, RXFP1 and RXFP2-expression. (DOC) [file pone.0039021.s003.doc]

| **No.** | **GSV** |  |  | **Varicose GSV** | | |  |
| --- | --- | --- | --- | --- | --- | --- | --- |
|  | Age [yr] | Gender |  | Age [yr] | Gender | |  |
| 1 | 66 | Female |  | 24 | Female | |  |
| 2 | 70 | Female |  | 25 | Female | |  |
| 3 | 71 | Female |  | 25 | Female | |  |
| 4 | 71 | Female |  | 29 | Female | |  |
| 5 | 74 | Female |  | 30 | Female | |  |
| 6 | 74 | Female |  | 30 | Female | |  |
| 7 | 77 | Female |  | 34 | Female | |  |
| 8 | 79 | Female |  | 38 | Female | |  |
| 9 | 81 | Female |  | 38 | Female | |  |
| 10 | 81 | Female |  | 40 | Female | |  |
| 11 | 58 | Male |  | 41 | Female | |  |
| 12 | 59 | Male |  | 42 | Female | |  |
| 13 | 61 | Male |  | 48 | Female | |  |
| 14 | 62 | Male |  | 55 | Female | |  |
| 15 | 66 | Male |  | 58 | Female | |  |
| 16 | 67 | Male |  | 61 | Female | |  |
| 17 | 68 | Male |  | 64 | Female | |  |
| 18 | 69 | Male |  | 65 | Female | |  |
| 19 | 70 | Male |  | 67 | Female | |  |
| 20 | 70 | Male |  | 67 | Female | |  |
| 21 | 71 | Male |  | 69 | Female | |  |
| 22 |  |  |  | 70 | Female | |  |
| 23 |  |  |  | 71 | Female | |  |
| 24 |  |  |  | 71 | Female | |  |
| 25 |  |  |  | 72 | Female | |  |
| 26 |  |  |  | 72 | Female | |  |
| 27 |  |  |  | 73 | Female | |  |
| 28 |  |  |  | 74 | Female | |  |
| 29 |  |  |  | 74 | Female | |  |
| 30 |  |  |  | 74 | Female | |  |
| 31 |  |  |  | 76 | Female | |  |
| 32 |  |  |  | 76 | Female | |  |
| 33 |  |  |  | 78 | Female | |  |
| 34 |  |  |  | 81 | Female | |  |
| 35 |  |  |  | 86 | Female | |  |
| 36 |  |  |  | 28 | Male | |  |
| 37 |  |  |  | 30 | Male | |  |
| 38 |  |  |  | 43 | Male | |  |
| 39 |  |  |  | 44 | Male | |  |
| 40 |  |  |  | 51 | Male | |  |
| 41 |  |  |  | 51 | Male | |  |
| 42 |  |  |  | 51 | Male | |  |
| 43 |  |  |  | 54 | Male | |  |
| 44 |  |  |  | 59 | Male | |  |
| 45 |  |  |  | 63 | Male | |  |
| 46 |  |  |  | 74 | Male | |  |
|  |  |  |  |  |  | |  |
| **Healthy GSV** | | | | **Varicose GSV** | | | |
|  | Female | Male | Total | Female | | Male | Total |
| No. | 10 | 11 | 21 | 35 | | 11 | 46 |
| Mean age | ~74 years | ~66 years | ~70 years | ~57 years | | ~ 50 years | ~ 53 years |

**Table S2**

Age and gender of the donor’s of healthy and varicose GSV samples used for immunohistochemical analysis of relaxin-2, RXFP1 and RXFP2-expression
